# The trivector approach for minimally invasive total knee arthroplasty: A technical note

**DOI:** 10.1007/s10195-012-0197-8

**Published:** 2012-04-17

**Authors:** Francesco Benazzo, Stefano Marco Paolo Rossi

**Affiliations:** Clinica Ortopedica e Traumatologica dell’Università degli Studi di Pavia, Fondazione IRCCS Policlinico San Matteo, P.le Golgi 19, 27100 Pavia, Italy

**Keywords:** Total knee replacement, Minimally invasive, Trivector

## Abstract

One of the main criticisms of minimally invasive approaches in total knee arthroplasty has been their poor adaptability in cases of major deformity or stiffness of the knee joint. When they are used in such cases, excessive soft-tissue tension is needed to provide appropriate joint exposure. Here, we describe the “mini trivector approach,” which has become our standard approach for total knee replacement because it permits us to enlarge the indication for minimally or less invasive total knee replacement to many knees where quad sparing, a subvastus approach, or a mini quad or mini midvastus snip may not be sufficient to achieve correct exposure. It consists of a limited double snip of the VMO and the quadriceps tendon that reduces tension on the extensor mechanism and allows easier verticalization of the patella as well as good joint exposure.

## Introduction

Since their introduction, one of the main criticisms of minimally invasive approaches in total knee arthroplasty has been their poor adaptability in cases of major deformity or stiffness of the knee joint. When used in such cases, excessive soft-tissue tension is needed to provide appropriate joint exposure, and the accuracy of implant positioning is reduced due to poor joint visualization.

This technical note presents the mini trivector approach for total knee arthroplasty as an alternative to classical minimally invasive approaches in patients with more difficult and stiff knees.

## Surgical technique

A curved incision with a lateral concavity medial to the midline of the joint is performed with the knee flexed at 90° to take advantage of the elasticity of the skin.

Incision is generally initiated 1 cm proximal to the superior pole of the patella and ends medial to the tibial tuberosity (TT). The length of the incision is determined by the dimensions of the patient and can be calculated preoperatively according to the mediolateral dimensions of the planned femoral component. In standard minimally invasive approaches such as mini midvastus snip or subvastus, the length of the incision is also determined by the thickness and mobility of the patella [1–3].

A parapatellar arthrotomy is performed ½ cm from the medial margin of the patella through the medial patellofemoral ligament, starting from the proximal pole of the patella and finishing at the TT.

**Fig. 1:**
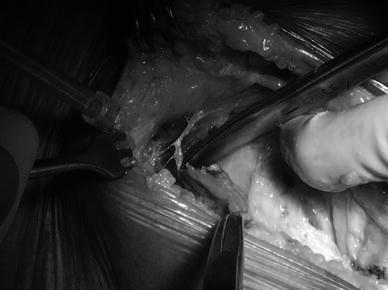
1.5 cm split of the vastus medialis obliquus (VMO) (left knee)

Once the arthrotomy has been performed, the vastus medialis must be correctly exposed, particularly its oblique fibers (vastus medialis obliquus or “VMO”) at the patellar insertion, and these are incised subcutaneously (with no violation of the skin above) along their direction for about 1.5 cm (Fig. 1).

This kind of approach (which is the classical mini midvastus snip) is sufficient in most cases to achieve good verticalization of the patella and correct joint exposure [4–6].

**Fig. 2:**
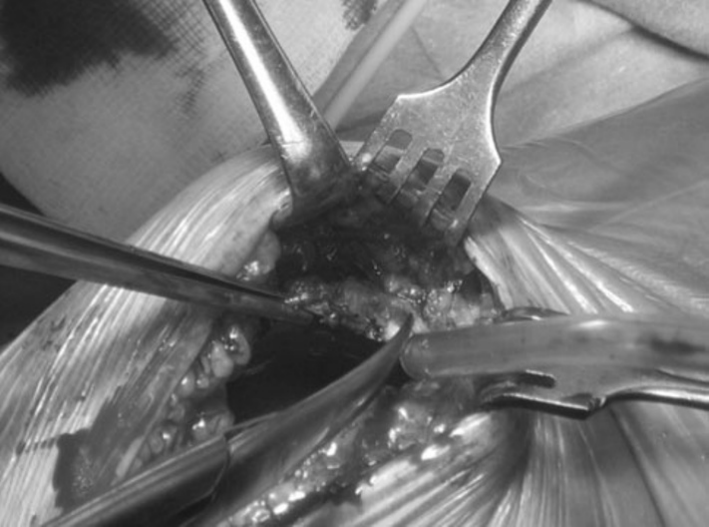
Mini trivector: 1 cm snip of the quadriceps tendon (right knee)

The trivector approach also includes a snip of the quadriceps tendon, continuing along the direction of the medial margin of the patella for approximately 1 cm (Fig. 2).

**Fig. 3:**
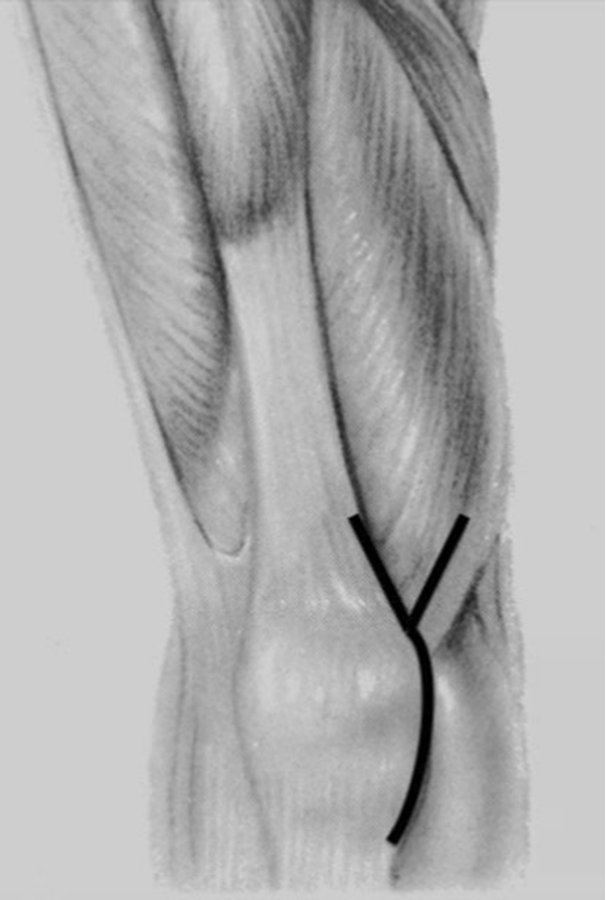
Mini trivector: schematic view of the approach (right knee)

Performing both snips enhances the mobility of the patella, especially when it is stiff or very thick, by reducing the tension on the extensor mechanism (Fig. 3).

The approach continues with subtotal removal of Hoffa’s body and subperiosteal detachment of the medial capsule from the proximal metaphysis of the tibia, respecting the tibial insertion of the medial collateral ligament (MCL), which permits good exposure of the medial tibial plateau.

At this point, the mobility of the patella is increased by removing the osteophytes: very often a lateral osteophyte does not allow sufficient movement and verticalization of the patella to achieve good exposure of the femur. Once verticalized, a preliminary cut of the patella is performed—either straight if a resurfacing is planned, or dihedral. The patellar cut gains space and lowers the tension on the extensor mechanism through easier lateral dislocation of the patella.

The presence of the two snips reduces the tension on the extensor mechanism and leads to a wider force distribution that avoids the possibility of the snips lengthening during flexion or hyperflexion of the knee.

## Indications

This kind of approach is very versatile, and can be used with all patterns of insertion of the VMO on the patella (low, medium, and high). It can be particularly useful with lower insertions of the VMO, considering that it reduces the tension on the extensor mechanism, which can be raised when trying to lateralize the patella.

More generally, this technique is suitable for all knees that are somewhat stiff or deformed but can still be operated on using a limited approach; it is not, however, indicated in cases where major exposure techniques are necessary (see the “Discussion” below).

## Discussion

One of the most important problems with the use of classical minimally invasive approaches in total knee arthroplasty is poor reproducibility due to the limited exposure they can provide, especially in stiff joints. This implies two main problems:

- Excessive tension on the soft tissues and in particular the extensor mechanism
- More difficult component positioning due to imperfect joint visualization [7].

These problems are usually solved after the learning curve is complete and good mastery of the “mobile window” concept is achieved [8].

It is true, on the other hand, that some stiff or severely degenerated knees with significant flexion contracture or very limited flexion are very hard to tackle with any of the classical minimally invasive approaches; there is a high risk that a 1.5 cm VMO snip performed in isolation will widen during the surgery due to the high tension placed on the extensor mechanism.

**Fig. 4:**
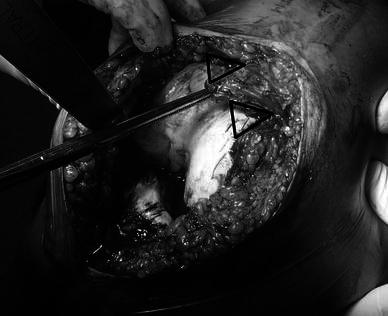
Mini trivector approach: the double triangle decreases the tension on the extensor mechanism (right knee)

In our experience, the minimally invasive approach known as the mini trivector approach leads to easier patellar verticalization and lateral dislocation, which are key to successfully performing a total knee arthroplasty with a minimally invasive approach, because the double limited snip enlarges the working space in the proximal operating field due to the double triangle it creates (Fig. 4), which allows better visualization of the joint on one side and reduces the forces on the muscular and tendon components on the other (Fig. 4).

The trivector approach allows us to release the extensor mechanism without performing major incisions on it and without compromising its function, which has been reported by Fischer et al. to occur with a standard medial trivector approach [9].

This approach has given us the ability to enlarge the indication for a minimally invasive approach to a wide range of cases that include “difficult” knees.

There are now very few cases where a minimally invasive approach is not suitable. Such cases involve:

- The need to perform a TT osteotomy
- The inability to move and verticalize the patella, even after correct preparation
- The inability to obtain correct exposure for the distal cut of the femur (fibrous ankylosis, very stiff knees).

In reference to the last two points, the trivector approach makes it possible to reduce the number of cases where these two issues become a contraindication for a minimally invasive approach.

Advantages of this technique include the possibility of approaching more difficult knees with limited exposure, the fact that the extensor mechanism is not compromised, and the good joint visualization available in order to achieve correct implant positioning.

On the other hand, being a minimally invasive approach, this technique cannot be used in cases of major deformity or stiffness that require an extensile approach.

## Conclusions

Minimally invasive surgery is a philosophy based on respecting soft tissues and particularly the extensor mechanism in order to allow patients to recover more easily and quickly, with a faster rehabilitation program [10].

The trivector approach described in this work provides a way to extend this kind of recovery program to a wider range of patients with more difficult knees while remaining on the safe side in terms of component positioning, the cementing phase, and preservation of the extensor mechanism.
